# Exotic *Eucalyptus* leaves are preferred over tougher native species but affect the growth and survival of shredders in an Atlantic Forest stream (Brazil)

**DOI:** 10.1371/journal.pone.0190743

**Published:** 2018-01-02

**Authors:** Walace P. Kiffer, Flavio Mendes, Cinthia G. Casotti, Larissa C. Costa, Marcelo S. Moretti

**Affiliations:** 1 Laboratory of Aquatic Insect Ecology, University of Vila Velha, Vila Velha, Brazil; 2 Graduate Program in Ecosystem Ecology, University of Vila Velha, Vila Velha, Brazil; BRAZIL

## Abstract

We evaluated the effect of leaves of native and exotic tree species on the feeding activity and performance of the larvae of *Triplectides gracilis*, a typical caddisfly shredder in Atlantic Forest streams. Leaves of four native species that differ in chemistry and toughness (*Hoffmannia dusenii*, *Miconia chartacea*, *Myrcia lineata* and *Styrax pohlii*) and the exotic *Eucalyptus globulus* were used to determine food preferences and rates of consumption, production of fine particulate organic matter (FPOM), growth and survival of shredders. We hypothesized that the consumption rates of leaves of *Eucalyptus* and their effects on the growth and survival of shredders could be predicted by leaf chemistry and toughness. The larvae preferred to feed on soft leaves (*H*. *dusenii* and *M*. *chartacea*) independently of the content of nutrients (N and P) and secondary compounds (total phenolics). When such leaves were absent, they preferred *E*. *globulus* and did not consume the tough leaves (*M*. *lineata* and *S*. *pohlii*). In monodietary experiments, leaf consumption and FPOM production differed among the studied leaves, and the values observed for the *E*. *globulus* treatments were intermediate between the soft and tough leaves. The larvae that fed on *H*. *dusenii* and *M*. *chartacea* grew constantly over five weeks, while those that fed on *E*. *globulus* lost biomass. Larval survival was higher on leaves of *H*. *dusenii*, *M*. *chartacea* and *S*. *pohlii* than on *E*. *globulus* and *M*. *lineata* leaves. Although *E*. *globulus* was preferred over tougher leaves, long-term consumption of leaves of the exotic species may affect the abundance of *T*. *gracilis* in the studied stream. Additionally, our results suggest that leaf toughness can be a determining factor for the behavior of shredders where low-quality leaves are abundant, as in several tropical streams.

## Introduction

In forest streams, allochthonous leaf litter is the main source of energy and carbon for heterotrophic organisms [1,2]. The leaf litter breakdown is a complex process, which involves physical, chemical and biological factors [3,4]. Regarding aquatic detritivores, invertebrate shredders are able to consume and convert leaf litter into biomass and fine particles of organic matter [5–7], and play a significant role in the decomposition process [8]. Therefore, the activity of these organisms contributes to the mineralization of organic matter and nutrient cycling and, consequently, to energy flow in stream ecosystems [9].

Studies conducted in tropical streams have suggested that the importance of shredders on leaf processing may vary across different regions [10–12]. While streams in tropical forests may have high values of abundance (10–27%) and biomass (4–54%) of shredders on the invertebrate fauna [13–15], streams in savannah-like ecosystems are known for the scarcity of these organisms [16,17]. Some authors proposed hierarchical models that distributed the sources of variability in the decomposition process at different spatial scales, ranging from leaf patches on the streambed to the characteristics of different regions and phytophysiognomies [12,18]. There are many local factors that may influence the role of shredders on leaf processing, such as the conservation level of riparian zones [15], water physical and chemical properties, water flow [19], leaf litter quality [20–23] and the presence of leaves of exotic species [24–27].

The intrinsic characteristics and the stage of decomposition of leaf litter determine its quality for consumers [28]. Therefore, the contents of lignin, cellulose, nutrients, and secondary compounds as well as microbial colonization influence leaf quality [29–31]. It is well known that invertebrate shredders exhibit a feeding preference for high-quality leaves, i.e., with high nutrient contents and low amounts of structural compounds [27,32,33]. When exposed to these leaves, shredders demonstrate high rates of consumption, growth and survival [34]. Consequently, high-quality leaves are processed more rapidly in streams [19,35].

Because of the high diversity of tree species in riparian zones [36,37], the leaf patches found on the streambed of tropical streams are heterogeneous [38,39]. However, changes in the riparian zones, such as the replacement of native forests by monocultures of exotic species [40,41], may alter the species composition of leaf patches and organic matter processing in streams [42–44]. Exotic tree species may have senescence periods that differ from those of native species [45,46], changing the quantity and quality of leaf litter in the streambed [47]. In addition, leaves of exotic species can present structural and chemical defenses that may limit the activity of invertebrate shredders [45,48]. Therefore, the presence of such leaves can affect shredder consumption rates and survival [49–51], promoting changes in the decomposition process and detritus-based food webs [40].

Different species of the genus *Eucalyptus* have been introduced worldwide [52,53]. When monocultures are created in the catchments of stream ecosystems, leaves of *Eucalyptus* can be found in leaf patches, mixed with those of native species [46,54]. Several studies have shown that the presence of these leaves influenced the feeding behavior of the shredders of temperate streams, which exhibited low consumption rates and high mortality [34,49,55]. In the tropics, some authors have suggested that the effect of *Eucalyptus* leaves might be less drastic in particular streams where invertebrate shredders are exposed to native leaves with low quality [41,56]. Therefore, it is expected that the behavior of the shredders of tropical streams could be more influenced by leaf characteristics than by their origin, i.e., leaves of native or exotic species [27].

The aim of this study was to evaluate the effect of leaves of native (*Hoffmannia dusenii* Standl., *Miconia chartacea* Triana, *Myrcia lineata* [O. Berg] Nied. and *Styrax pohlii* A. DC.) and exotic tree species (*Eucalyptus globulus* Labill.) on the feeding activity and performance of larvae of *Triplectides gracilis* (Burmeister, 1839), a typical caddisfly shredder in Atlantic Forest streams. We determined the food preferences and rates of consumption, production of fine particulate organic matter (FPOM), growth and, survival of *T*. *gracilis* in laboratory. We hypothesized that the consumption rates of leaves of *Eucalyptus* and their effects on the growth and survival of shredders could be predicted by leaf chemistry and toughness.

## Materials and methods

### Study area

The shredders and leaves used in this study were collected in Macuco Stream (20°01′23.1" S– 40°32′58.6" W), located at 593 m a.s.l. on the edge of an Atlantic Forest fragment in the municipality of Santa Leopoldina, Espírito Santo (SE Brazil). The riparian vegetation of the studied reach is well-developed, shading approximately 80% of the streambed. However, a monoculture of *E*. *globulus* and a small rural property are located close to one of stream margins. The streambed is heterogeneous, with the presence of pebbles, gravel and leaf patches. During the study period, the stream water was slightly acid to neutral (pH: 6.5–7.2), well oxygenated (7.15–8.23 mg L^-1^), with temperature and conductivity ranging between 18.1–19.5°C and 22.8–29.3 μS cm^-1^, respectively. More information about Macuco Stream is available in a previous study [15].

### Leaves and shredders

The four native leaves used in this study (*H*. *dusenii* [Rubiaceae], *M*. *chartacea* [Melastomataceae], *M*. *lineata* [Myrtaceae] and *S*. *pohlii* [Styracaceae]) are among the most abundant of the vertical inputs of leaf litter measured over the course of a year in the studied reach. Leaves of these species were chosen because they differ in chemistry and toughness. The leaves of the exotic species *E*. *globulus* (Myrtaceae) were included in the experiments because they are found year-round on the streambed of the studied reach. Leaves of the five species were collected using eight litter traps (1 m^2^, 10 mm mesh) that were set at 1.5 m above the ground on both stream margins, in a 50 m long reach. During two months, these traps were checked every 15 days, and the leaves were taken to the laboratory, where they were dried at room temperature, sorted and stored separately until the start of the experiments.

Larvae of *T*. *gracilis* (Trichoptera, Leptoceridae; 2^nd^–3^rd^ instars) were collected manually from pools in the studied reach. In these areas, the larvae of *T*. *gracilis* are easily found on leaf patches. The collected larvae were placed in coolers containing stream water and were transported on the same day to the laboratory, where they were starved for 48 h under a constant photoperiod (12 L: 12 D), temperature (21°C) and aeration. New larvae were collected for each block of replicates in the laboratory trials described below. Leaves and shredders were collected in accordance with federal laws and the regulations of the Brazilian Environmental Ministry. The sampling site was private, and permission from the owner was obtained prior to sampling. The studied species were not protected by Brazilian law or red-listed.

### Leaf characterization

Prior to the start of the laboratory trials, the leaves of each species were placed separately in fine mesh bags (10 × 15 cm, 0.5 mm mesh) and conditioned for 15 days in Macuco Stream. After conditioning, the leaves of four bags of each species were used for the determination of leaf characteristics. The leaves of each bag were oven-dried (60°C, 72 h), ground in an electrical mill and homogenized. Samples of 100 mg were used to determine the contents of total phenolics [57], nitrogen and phosphorus [58]. The contents of lignin and cellulose were determined from 250 mg samples by the gravimetric method [59]. Leaf toughness was measured indirectly by the force required to tear apart a leaf sample [60]. All analyses were done in four replicates.

### Food preference experiment

**Fig 1:**
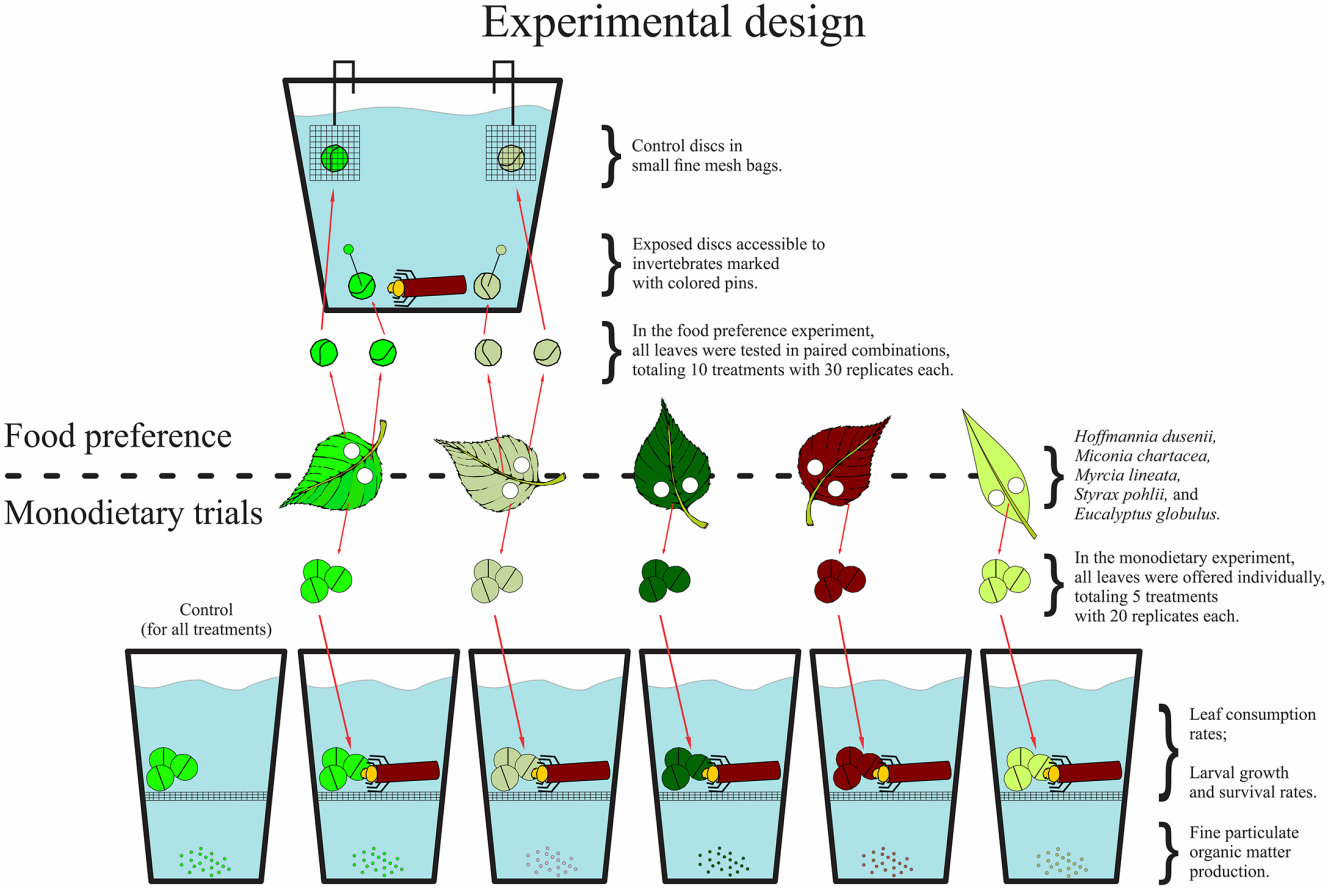
Experimental design of the laboratory trials set to evaluate the feeding activity and performance of larvae of *Triplectides gracilis* exposed to leaves of *Hoffmannia dusenii*, *Miconia chartacea*, *Eucalyptus globulus*, *Myrcia lineata*, and *Styrax pohlii*.

The food preference experiment followed a classic experimental design [21]. Leaf discs (14 mm diameter) were cut from the conditioned leaves and offered to shredders in all possible pairwise comparisons (10 treatments in total). Larvae of *T*. *gracilis* of similar size (initial biomass: 2.52 ± 0.35 mg, mean ± SE) were placed individually in plastic cups (9 cm diameter and 13 cm height) containing 400 ml of the filtered stream water. The replicates were maintained under the same photoperiod and temperature described above. Pairs of discs were cut from contiguous areas of the leaves, avoiding the main vein, so that we could assume that the discs of each pair had the same initial mass. One disc of each pair was offered to the larvae (exposed disc), while the other was placed in a small fine mesh bag (0.5 mm) and fixed to the edge of the cup, so that these discs were submerged but inaccessible to shredders (control discs; Fig 1). The exposed discs of the different species were pierced with colored pins to facilitate their identification. Thirty replicates of each treatment were established, and the whole experiment was performed in 3 blocks containing 10 replicates of each treatment. The replicates were discontinued when at least two-thirds of the exposed discs had been consumed or after 10 days.

The remaining leaf material (exposed and control discs) and larvae were dried (60°C, 72 h) and weighed (0.1 mg). The consumption of each species was expressed in terms of mg of leaf material consumed during the experiment and was measured as the difference between the mass of the control and exposed discs (mg) divided by the larval biomass (mg) and the feeding period (days).

### Monodietary experiment

A total of 100 larvae of *T*. *gracilis* (initial biomass: 2.12 ± 0.21 mg) were used in this experiment. The larvae were individually placed in cups similar to those described above, but with the bottom covered by fine mesh (1.0 mm) to keep the FPOM produced inaccessible to shredders. Three leaf discs from the same species, for which the initial mass had already been measured, were offered in each cup, and the larvae were allowed to feed *ad libitum* throughout the experiment (Fig 1). For each treatment (leaves of one species), 20 replicates were established. Five additional replicates of each treatment that contained no larvae (controls) were established to determine the disc mass loss in the absence of shredders and to correct the mass of the remaining discs. The monodietary experiment lasted five weeks, and the discs and water of each replicate were replaced every week. The remaining discs were oven-dried (60°C, 72 h) and weighed (0.01 mg). The initial dry mass of the offered discs was determined using a wet mass/dry mass correction factor that was previously calculated for each species. The consumption rates were calculated weekly and expressed in terms of mg of leaf consumed (difference between the initial and final mass of the discs) divided by the larval biomass (mg) and the feeding period (7 days).

To determine the rates of FPOM production, the water in each replicate was filtered over pre-weighed glass fiber filters (GF-3, 0.6 μm, 47 mm, Macherey-Nagel, Germany). The filters were oven-dried (60°C, 72 h) and weighed as described above. The FPOM production rates were calculated weekly for each treatment and expressed in terms of mg of FPOM produced (difference between the initial and final mass of the filters) divided by the larval biomass (mg) and the feeding period (7 days).

The approximate digestibility of the leaves in each treatment was used to evaluate the proportion of organic material consumed that was processed and absorbed by the larvae [61]. This proportion was calculated weekly and expressed as the difference between the amount of leaf consumed and the FPOM produced (mg) divided by the amount of leaf consumed (mg).

The growth rates of *T*. *gracilis* were determined by the increase in larval biomass. The biomass of each larva was determined indirectly through a size-mass equation that was determined previously for the studied population. This equation uses the tibia length as a predictor of biomass [62]:

$$\text{ln}\;\text{DM}=1.60\;\text{TL}–1.38\left({\text{r}}^{2}=0.80\right)$$

where DM is the dry mass of a larva (mg), and TL is the length of the two segments of the tibia from the third left leg (mm). Every week, the larvae were individually photographed, and the tibia length was measured using ImageJ Software (US National Institutes of Health, Bethesda, Maryland, USA; 0.1 mm). Larval growth was expressed as the weekly change in larval biomass (mg), which was calculated by the difference in larval biomass in each week of the experiment.

During the experiment, the number of living larvae in each treatment was determined daily for the calculation of survival rates, which were expressed as the percentage of the initial number of larvae that were alive in each day of the experiment. The replicates in which the larvae pupated (maximum of 3 per treatment) were not considered.

### Data analysis

Analyses of variance (ANOVA) were used to compare the values of leaf chemistry and toughness of the studied leaves. Percent data was transformed (arcsine [√x]) to fit a normal distribution. Tukey tests were used for *post hoc* comparisons. In the food preference experiments, the data analysis was performed in two steps [63]. First, we determined whether the larvae had consumed a significant amount of the exposed discs. In each treatment, a species was considered “consumed” when the average mass of the exposed discs was significantly lower than the corresponding control discs (paired *t*-tests; [64]). Second, the consumption of the exposed discs in each treatment was also compared by paired *t*-tests to determine whether the larvae preferred either of the two offered species.

In the monodietary experiments, the leaf consumption, FPOM production and larval growth data were compared among leaf treatments and feeding periods (weeks) using Analysis of Variance with repeated measures (RM-ANOVA), followed by Tukey tests. The proportions of approximate digestibility were compared by the non-parametric Kruskal-Wallis test followed by Dunn’s test. The survival of the larvae in the different treatments were compared by log-rank tests [65]. The normality and homogeneity of variance were tested for all data, and ANOVA models were validated by analysis of residuals. The statistical analyses were performed in Statistica 7 software (StatSoft Inc., Tulsa, USA).

## Results

### Leaf characterization

The leaves of *H*. *dusenii* had the lowest values of toughness and the highest content of nitrogen (Table 1). The second lowest values of toughness and the highest content of phenolics were found for *M*. *chartacea*. The leaves of *E*. *globulus* had the lowest content of nitrogen and intermediate values of toughness, while *S*. *pohlii* and *M*. *lineata* had the highest content of lignin and cellulose and high values of toughness. Phosphorus was similar among all studied species.

**Table 1:** Leaf chemistry and toughness values for the leaves of *Hoffmannia dusenii*, *Miconia chartacea*, *Eucalyptus globulus*, *Myrcia lineata*, and *Styrax pohlii* conditioned for 15 days in Macuco Stream.

Species	Total phenolics (%)	N (%)	P (%)	Cellulose (%)	Lignin (%)	Toughness (g)
*Hoffmannia dusenii*	N.D.	3.04 ± 0.12^a^	0.023 ± 0.001^a^	24.55 ± 0.45^a^	11.89 ± 0.24^a^	60.91 ± 16.04^a^
*Miconia chartacea*	0.91 ± 0.01^a^	1.58 ± 0.18^b^	0.020 ± 0.000^a^	21.29 ± 3.05^a^	24.81 ± 4.83^b^	142.68 ± 10.74^a^
*Eucalyptus globulus*	0.43 ± 0.21^bc^	0.90 ± 0.05^d^	0.020 ± 0.000^a^	33.34 ± 0.78^b^	24.10 ± 4.06^b^	201.62 ± 41.35^b^
*Myrcia lineata*	0.19 ± 0.00^c^	1.35 ± 0.15^c^	0.020 ± 0.000^a^	33.62 ± 0.55^b^	28.57 ± 4.52^b^	512.13 ± 33.48^c^
*Styrax pohlii*	0.75 ± 0.01^ab^	1.31 ± 0.28^c^	0.027 ± 0.002^a^	6.57 ± 0.20^c^	57.77 ± 5.94^c^	633.20 ± 47.27^c^
Variation (%)	379	238	35	412	386	939
df	(3,12)	(4,15)	(4,15)	(4,15)	(4,15)	(4,15)
F	8.91	1579.76	2.24	58.42	58.93	56.63
p	0.002	< 0.001	0.13	< 0.001	< 0.001	< 0.001

Mean ± SE. n = 4. F and p values are results from Analysis of Variance (ANOVA). N.D. = values under the detection limit. Values with different superscript letters differed significantly.

### Food preference experiment

**Fig 2:**
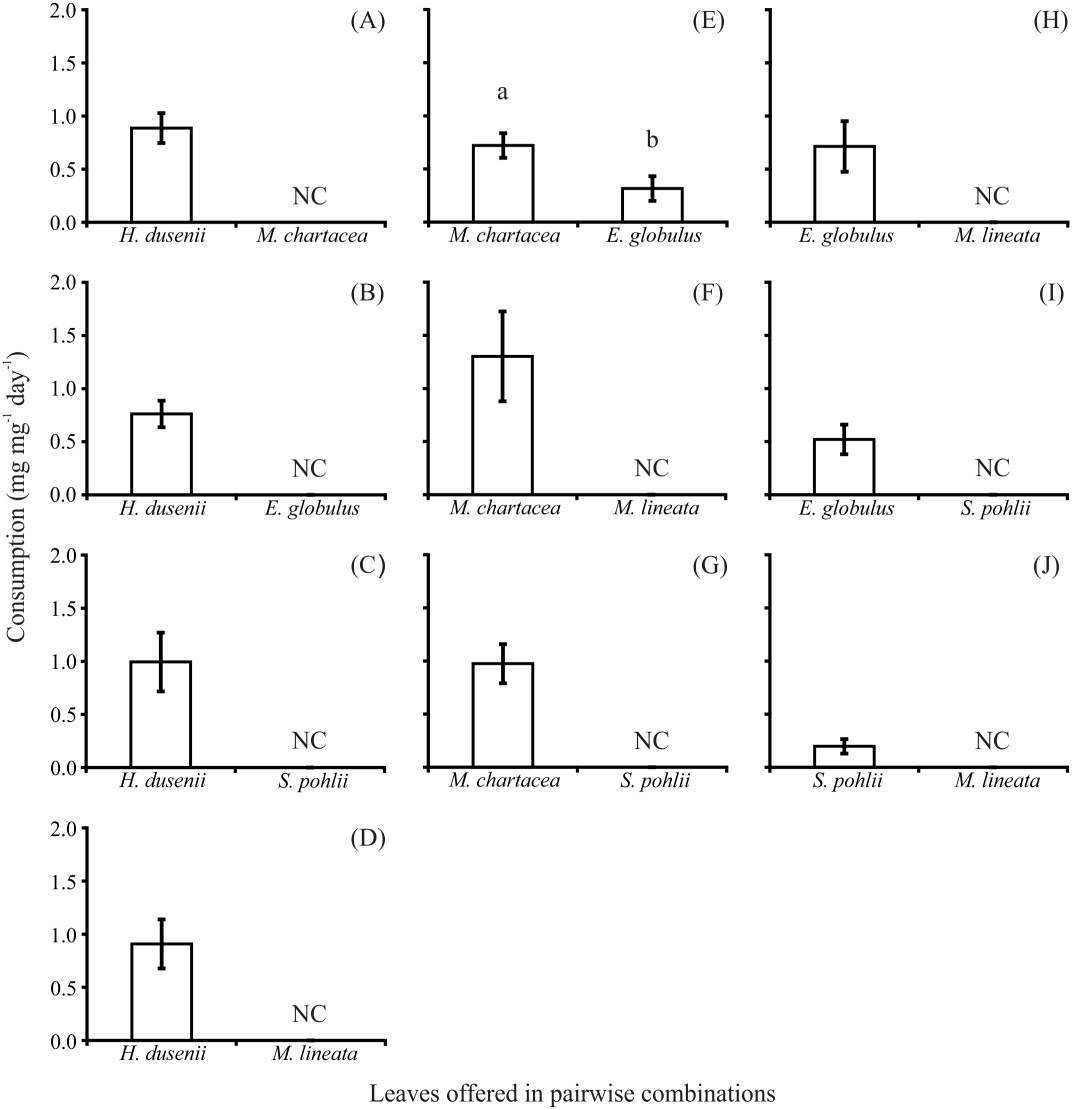
Consumption rates (mean ± SE) of larvae of *Triplectides gracilis* exposed to leaves of *Hoffmannia dusenii*, *Miconia chartacea*, *Eucalyptus globulus*, *Myrcia lineata*, and *Styrax pohlii* in paired combinations. Each graphic (A–J) presents the results of one paired combination (treatment). NC = Not consumed by larvae. Values with different superscript letters differed significantly. n = 30 (per treatment). Experiment duration = 10 days.

In the food preference experiment, *H*. *dusenii* was preferred in all pairwise treatments in which it was offered to shredders (0.76 ± 0.13 to 0.99 ± 0.28 mg mg^-1^ day^-1^), and none of the other species was consumed in its presence (Fig 2). The second most preferred species was *M*. *chartacea* (0.72 ± 0.16 to 1.30 ± 0.42 mg mg^-1^ day^-1^). Although the larvae of *T*. *gracilis* also consumed *E*. *globulus* in the presence of *M*. *chartacea*, the native species was preferred (*t*_*58*_ = -2.02, p = 0.040). In treatments in which *H*. *dusenii* and *M*. *chartacea* were absent, the larvae preferred *E*. *globulus* (0.52 ± 0.14 to 0.71 ± 0.24 mg mg^-1^ day^-1^) and did not consume *M*. *lineata* or *S*. *pohlii*. In the treatment in which *M*. *lineata* and *S*. *pohlii* were offered together, the larvae preferred *S*. *pohlii* but consumed overall very little (0.20 ± 0.07 mg mg^-1^ day^-1^).

### Monodietary experiment

**Fig 3:**
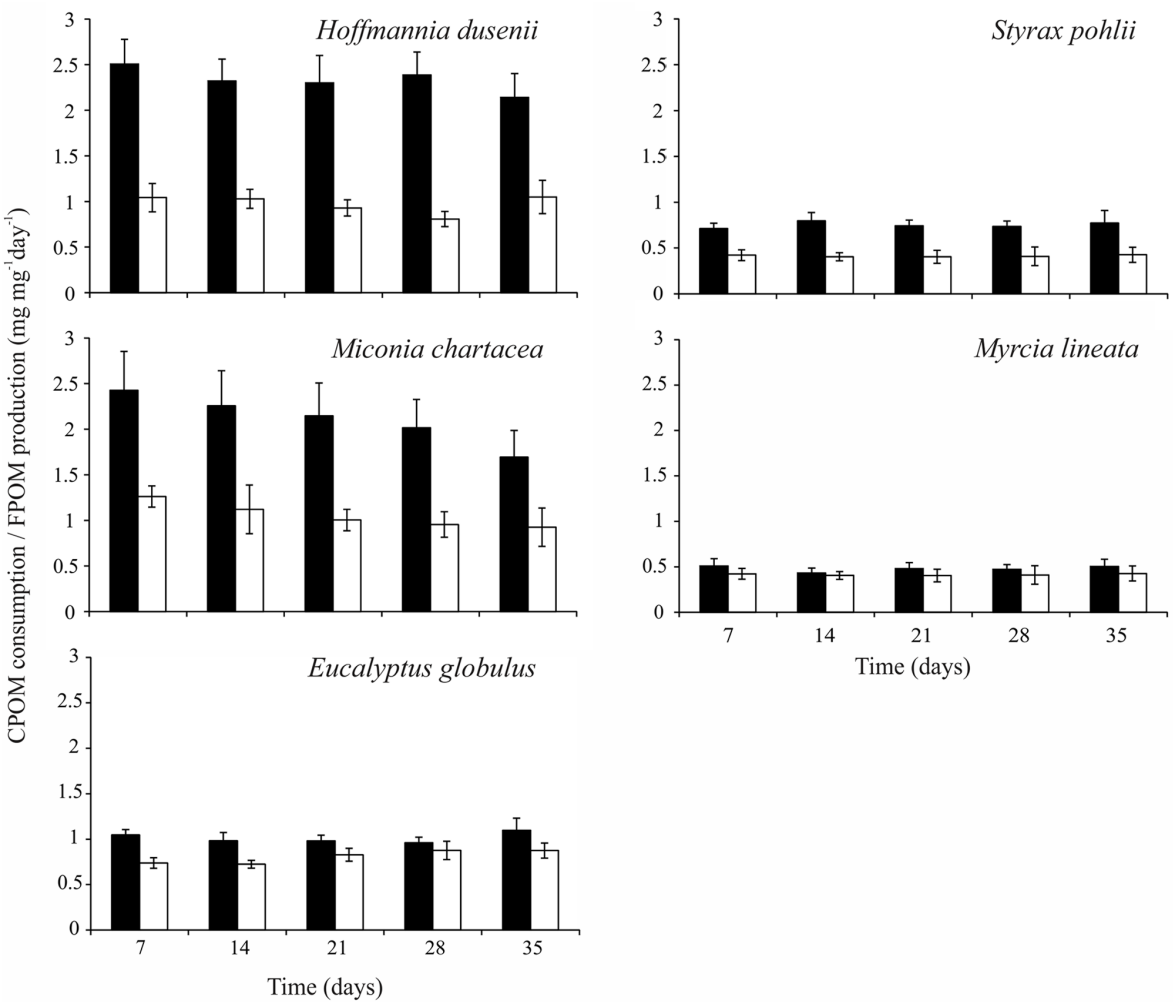
Consumption rates (black bars, mean ± SE) and production of fine particulate organic matter (white bars, mean ± SE) of larvae of *Triplectides gracilis* fed on leaves of *Hoffmannia dusenii*, *Miconia chartacea*, *Eucalyptus globulus*, *Myrcia lineata*, and *Styrax pohlii* in monodietary experiments. n = 20 (per treatment). Experiment duration = five weeks.

In the monodietary experiment, the larvae of *T*. *gracilis* exhibited high consumption rates in the treatments containing *H*. *dusenii* (2.34 ± 0.11 mg mg^-1^ day^-1^) and *M*. *chartacea* (2.12 ± 0.16 mg mg^-1^ day^-1^; Fig 3). Furthermore, high rates of FPOM production were observed when the larvae fed on these species (*H*. *dusenii*: 0.97 ± 0.05 mg mg^-1^ day^-1^; *M*. *chartacea*: 1.06 ± 0.07 mg mg^-1^ day^-1^), and these values were 2 times higher than those in other treatments. The larvae exposed to *E*. *globulus* exhibited intermediate rates of leaf consumption and FPOM production (1.00 ± 0.06 and 0.79 ± 0.06 mg mg^-1^ day^-1^, respectively), while low rates were observed in the treatments containing *S*. *pohlii* and *M*. *lineata* (consumption: 0.53 ± 0.08 and 0.47 ± 0.03 mg mg^-1^ day^-1^; FPOM production: 0.41 ± 0.03 and 0.36 ± 0.02 mg mg^-1^ day^-1^). The rates of leaf consumption and FPOM production differed among the treatments (Table 2). The proportions of approximate digestibility also differed among the treatments (H = 36.27; p < 0.01), and higher values were observed when the larvae fed on *H*. *dusenii*, *S*. *pohlii* and *M*. *chartacea* (median [interquartile range]: 0.54 [0.35], 0.51 [0.62] and 0.46 [0.26], respectively) than on *M*. *lineata* and *E*. *globulus* (0.20 [0.24] and 0.23 [0.26], respectively).

**Table 2:** Variation in the rates of leaf consumption, FPOM production and growth of larvae of *Triplectides gracilis* in monodietary experiments depending on leaves (studied species) tested by Analysis of Variance with repeated measures.

	df	SS	F	p
**Leaf consumption rate**				
*Leaves*	4	180.36	12.41	<0.001
*Error*	56	203.41		
**FPOM production rate**				
*Leaves*	4	26.12	10.70	<0.001
*Error*	56	34.14		
**Growth rates**				
*Leaves*	4	244.48	18.45	<0.001
*Error*	56	182.16		

df = degrees of freedom. SS = sum of squares.

**Fig 4:**
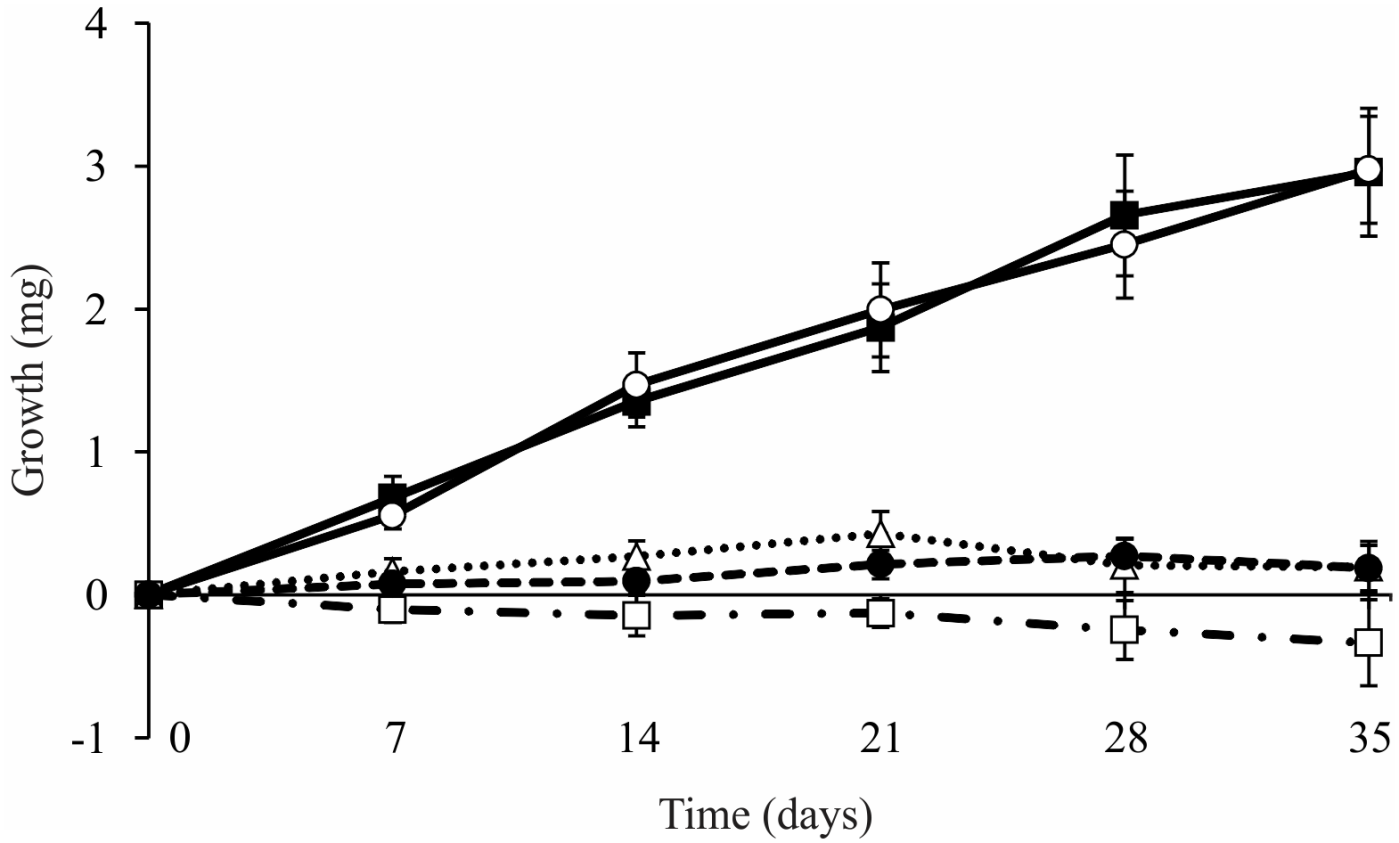
Growth rates (mean ± SE) of larvae of *Triplectides gracilis* fed on leaves of (○) *Hoffmannia dusenii*, (■) *Miconia chartacea*, (□) *Eucalyptus globulus*, (△) *Styrax pohlii*, and (●) *Myrcia lineata* in monodietary experiments. n = 20 (per treatment). Experiment duration = five weeks.

Constant growth rates were observed when the larvae of *T*. *gracilis* fed on *H*. *dusenii* and *M*. *chartacea* (0.59 ± 0.08 mg week^-1^ for both). After five weeks, the larvae in these treatments had approximately doubled their initial biomass (Fig 4 and Table 2). The larvae that fed on *S*. *pohlii* and *M*. *lineata* maintained their initial biomass, while the larvae that fed on *E*. *globulus* had negative growth rates (-0.06 ± 0.02 mg week^-1^), i.e., they lost biomass during the experiment.

**Fig 5:**
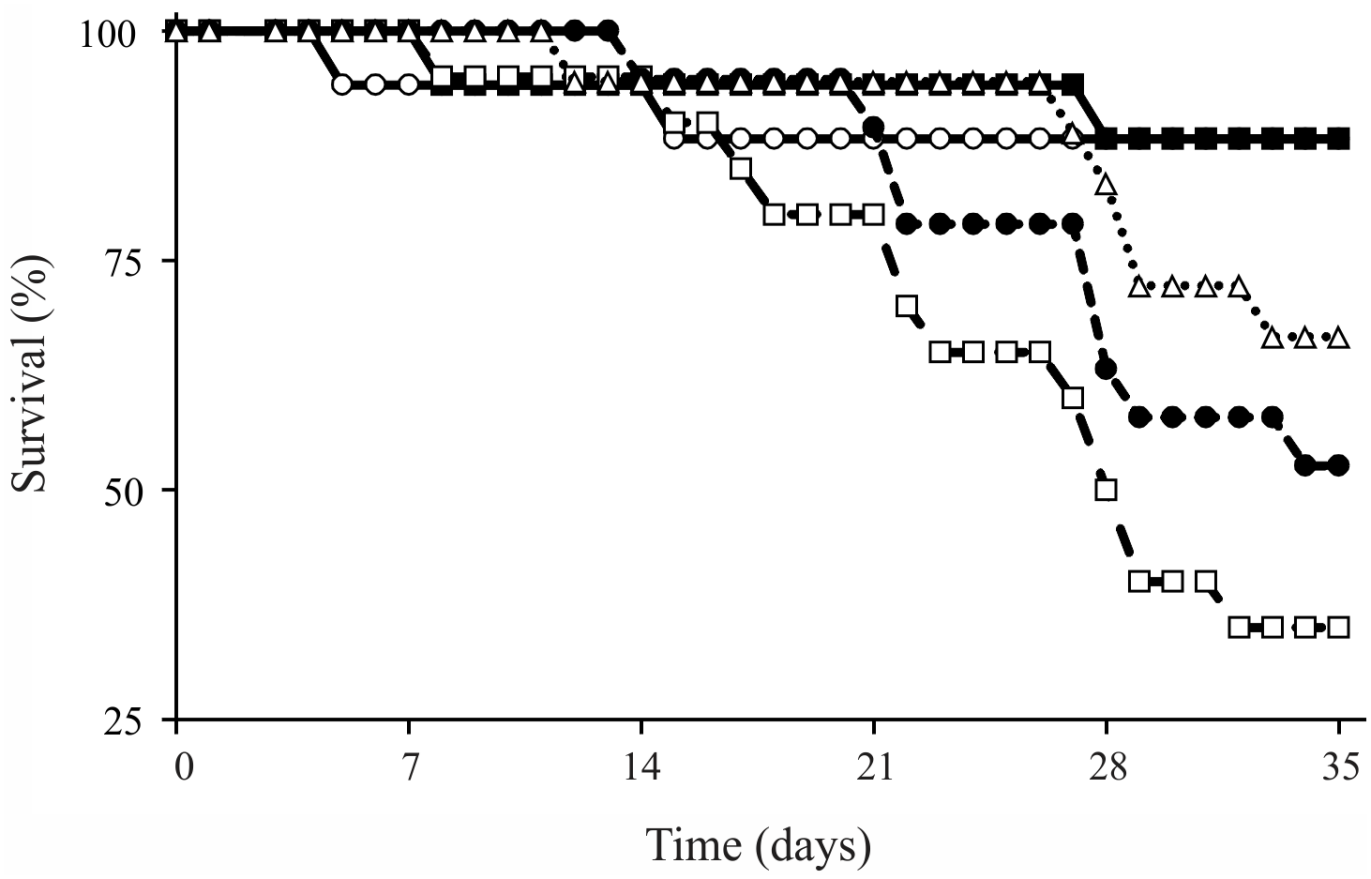
Survival rates of larvae of *Triplectides gracilis* fed on leaves of (○) *Hoffmannia dusenii*^a^, (■) *Miconia chartacea*^a^, (□) *Eucalyptus globulus*^b^, (△) *Styrax pohlii*^a^, and (●) *Myrcia lineata*^b^ in monodietary experiments. n = 20 (per treatment). Experiment duration = five weeks. Species with different superscript letters differed significantly.

At the end of the experiment, the average survival rate of larvae across all treatments was 66%. The larvae that fed on *E*. *globulus* and *M*. *lineata* had low survival rates (35 and 52%, respectively), and these values differed of those observed in the other treatments (log-rank test > -3.00, p < 0.01; Fig 5). The survival rates of the larvae that fed on *H*. *dusenii*, *M*. *chartacea* and *S*. *pohlii* were high (88, 88 and 72%, respectively) and did not differ from one another (p > 0.25, for all comparisons).

## Discussion

The leaves used in this study were chosen because they differ in chemistry and toughness. In spite of the values of lignin and cellulose had wider ranges than the contents of nitrogen and phenolic compounds, the variation of these leaf characteristics did not differ substantially. The content of phosphorus, which is considered a limiting nutrient for invertebrate consumption and growth [66], was the only leaf characteristic that did not differ among the leaves, and the observed values were consistent with those normally found in species from tropical rainforests [67]. While softer leaves had values of toughness that were similar to those normally found in temperate species [21,23,68], the values observed in the tough leaves are among the highest available in the literature [69,70]. Therefore, in our laboratory trials shredders were exposed to leaves that together formed a gradient of toughness. Because the leaves of *E*. *globulus* were intermediate in toughness, we predicted that larvae of *T*. *gracilis* would prefer and consume higher amounts of leaves of the exotic species than tougher native species (*M*. *lineata* and *S*. *pohlii*).

Leaf consumption in the food preference experiment varied among species, and high consumption rates were observed when the larvae fed on the soft leaves. These results are most likely related to the difficulty in consuming tough leaves for invertebrate shredders [70]. Leaves with high values of toughness mainly affect the initial instars of insect shredders because of the fragility of their mouthparts [21,26,71]. Moreover, the high content of structural compounds may have limited the growth and activity of microorganisms during leaf conditioning [3], decreasing the influence of these decomposers on leaf quality and palatability to shredders [72].

According to the patterns observed in the food preference experiment, the rates of leaf consumption and FPOM production in the monodietary treatment containing the leaves of *E*. *globulus* were intermediate. Although these rates differed among the studied species, the feeding activity of the larvae of *T*. *gracilis* could not be predicted by the contents of nutrients and phenolic compounds. The low amounts of phenolic compounds observed in the studied leaves could be related to the leaching process during conditioning, which tends to be faster in streams with warm temperatures [73,74]. Studies conducted with tropical leaves have found no influence of the content of secondary compounds on decomposition rates and the activity of invertebrate shredders [27,74,75]. Because of the low quality of leaf litter available on the streambed of tropical streams, the influence of leaf toughness on the food preferences and consumption rates of invertebrate shredders [21,71] and on the decomposition process [76,77] appears to be more important than the contents of nutrients and secondary compounds [22,74,78].

The amount of food ingested by a particular consumer is normally related to its nutritional value [79,80]. Although the leaves of *E*. *globulus* had the lowest N content, the consumption rates of the larvae feeding on this species were higher than those observed in treatments containing leaves with high nutrient content (*M*. *lineata* and *S*. *pohlii*). These results suggest that, when exposed to leaves of *E*. *globulus*, the larvae of *T*. *gracilis* may have compensated for the low quality of this diet by increasing their consumption rates. Through compensatory feeding, consumers may obtain the required amounts of nutrients even when environmental conditions restrict the quality of the available food resources [81]. However, this strategy can affect growth rates and survival over time [82], as observed in this study.

In spite of the rates of FPOM production being related to leaf consumption in all treatments, the leaves of *M*. *lineata* and *E*. *globulus* had the lowest proportions of approximate digestibility, i.e., the larvae did not digest a significant amount of the food ingested. The leaves of tree species of Myrtaceae are characterized by having a high number of essential oil glands [83,84]. Larvae of *Tipula lateralis* Meigen, 1830 (Diptera, Tipulidae) fed with leaves of *E*. *globulus* experienced damage to their mouthparts and changes in their gut microbiota [50]. Moreover, the essential oils caused a 25% reduction in the enzymatic and absorption capacities of these shredders. Because of their antifungal activity [48,50], the oils produced by the leaves of *Eucalyptus* may also alter the activity of decomposing fungi [48] and, consequently, leaf conditioning [45] and quality [74].

The growth rates and survival of shredders exposed to native species followed the patterns observed in the food preference experiment. The larvae that fed on soft leaves (*H*. *dusenii* and *M*. *chartacea*) increased in mass and had greater survival. When exposed to tough leaves (*M*. *lineata* and *S*. *pohlii*), the larvae of *T*. *gracilis* had low consumption rates and did not increase in mass over five weeks. However, the survival of the larvae fed with *S*. *pohlii* was higher than those fed with *M*. *lineata*, not differing from values observed in the treatments containing soft leaves. These results are most likely related to the proportions of approximate digestibility of the studied leaves, which indicated that larvae fed on *H*. *dusenii*, *S*. *pohlii* and *M*. *chartacea* assimilated large amounts of ingested food [85,86].

Contrary to shredders food preferences, the effect of the leaves of *E*. *globulus* on the growth rates and survival of *T*. *gracilis* could not be predicted. When feeding on this species for several days, the larvae lost mass and had low survival. Because the survival rates decreased considerably from the third week of the experiment, our results suggest that the leaves of *E*. *globulus* could not provide all of the nutrients required to maintain the metabolism and growth of the larvae of *T*. *gracilis* [50]. Beyond the low nutritional quality, these leaves contain compounds that may be toxic to consumers [34,48]. Some herbivorous insects may not detect the presence of toxic compounds when exposed to a new food item and may consume significant amounts of this resource, even when growth rates and survival are affected [87,88].

The obtained results demonstrate that the effects of the leaves of *E*. *globulus* on the feeding behavior of the larvae of *T*. *gracilis* could be predicted by leaf toughness. However, the long-term consumption of these leaves negatively influenced shredder growth and survival. Although the leaves of *E*. *globulus* were preferred over tougher native species, the consumption of these leaves for long periods may affect the abundance of *T*. *gracilis* in the studied stream. Furthermore, because the larval food preferences and consumption rates were not substantially influenced by the content of nutrients and secondary compounds, our results suggest that the values of leaf toughness and the content of structural compounds can be determining factors for the behavior and activity of shredders in ecosystems where low-quality leaves are abundant, as in several tropical streams. The observed patterns can also vary across regions because the genus *Eucalyptus* has several species and hybrids that present different contents of lignin and cellulose, which are related to leaf toughness. In addition, it is known the effect of *Eucalyptus* on the preference and performance of shredders differs in streams where this genus naturally occurs in the riparian vegetation from those where it was introduced and contributes with low quality leaves. Overall, this study reinforces the need to better assess the effects caused by the introduction of *Eucalyptus* in the surroundings of lotic environments, given that the presence of these leaves on the streambed can influence the detritus-based food chains and energy flow in these ecosystems.

## Supporting information

S1 File Leaf characteristics data. Leaf chemistry and toughness values for the leaves of *Hoffmannia dusenii*, *Miconia chartacea*, *Eucalyptus globulus*, *Myrcia lineata*, and *Styrax pohlii* conditioned for 15 days in Macuco Stream.
(XLSX)

S2 File Food preference data. Dry mass values of control and exposed discs cut from leaves of *Hoffmannia dusenii*, *Miconia chartacea*, *Eucalyptus globulus*, *Myrcia lineata*, and *Styrax pohlii* and offered to shredders in all possible pairwise comparisons.
(XLSX)

S3 File Monodietary experiment data. Consumption rates, production of fine particulate organic matter, growth, and survival of larvae of *Triplectides gracilis* fed on leaves of *Hoffmannia dusenii*, *Miconia chartacea*, *Eucalyptus globulus*, *Myrcia lineata*, and *Styrax pohlii* in monodietary experiments.
(XLSX)
